# Nightingale: A multimodal approach combining EEG signals and audio features to enhance music therapy

**DOI:** 10.1371/journal.pone.0341497

**Published:** 2026-01-27

**Authors:** Zirui Chen, Nikhil Yadav

**Affiliations:** 1 The Webb Schools, Claremont, California, United States of America; 2 Department of Computer Science, Shiley-Marcos School of Engineering, University of San Diego, San Diego, California, United States of America; Georgia State University, UNITED STATES OF AMERICA

## Abstract

Music therapy has emerged as a promising, yet underutilized, treatment modality in various clinical settings. This paper presents Nightingale, a novel multimodal approach that integrates audio features with electroencephalogram (EEG) signals to predict a subject’s emotional response to music. The accuracy of emotional response predictions is enhanced using audio features coupled with EEG data extracted from the Dataset for Emotion Analysis using Physiological and Audiovisual Signals (DEAP). These combined modalities are used to develop a Multilayer Perceptron and Convolutional Neural Network architecture, achieving a Mean Absolute Percentage Error (MAPE) values of 19.52 for valence, 22.16 for arousal, and an R squared of 0.67 for the two domains. This approach offers superior performance to the most recent state-of-the-art in the field. Additionally, it requires significantly less computational resources and a simple network structure, providing an evidence-based prediction technique that could make music therapy more effective and accessible.

## Introduction

### Background

Music therapy has consistently demonstrated its potential across various clinical applications. It has been shown to reduce anxiety, alleviate mood, and improve depressive symptoms, offering a viable alternative to conventional treatment methods [1]. The scope of music therapy spans fields such as dementia care, palliative medicine, and autism spectrum disorder (ASD) [2–4]. It serves diverse populations, including veterans, patients, their families, and the public [5–8]. However, due to the high costs associated with therapy sessions and the shortage of certified music therapists, music therapy remains largely inaccessible to the broader population. Therefore, this study aims to address this accessibility gap by presenting a novel, efficient method of music emotion recognition to enhance therapeutic effects.

### History of field

The field of Music Emotion Recognition (MER) has seen various approaches, making it challenging to compare the results between different studies. However, a common approach to quantifying emotion induced by music is to use the valence-arousal system, where valence represents positivity of emotion and arousal represents intensity of emotion [9].

Some researchers have focused on emotion recognition through lyrics and tags, exemplified by the Affective Norms for English Words (ANEW) database, which provides pleasure, arousal, and dominance (PAD) labels for English words [10]. This method leverages the more established field of Natural Language Processing (NLP). However, given the rapid expansion of digital music libraries and the limitations of neglecting the audio content itself, such methods lack scalability.

Subsequent research has focused more on audio content, identifying features that best inform emotional responses. Features like Mel-Frequency Cepstral Coefficients (MFCCs), Zero Crossing Rate (ZCR), voice quality, fundamental frequency (F0), F0 envelope, Linear Spectral Pairs (LSP), and intensity and loudness features, have been used by researchers in this area.

### Advantage of multimodality

Many works in the 2010s have shown progress in this area. Despite these advances, the MER community believes that there is an upper limit to the accuracy of models using only acoustic features, as they fail to account for non-audio factors, such as social contexts [11]. This has led to a growing interest in multimodal approaches that combine multiple feature domains. Researchers have experimented with various fusions, such as audio and lyrics or audio and tags, consistently finding that multimodal models outperform unimodal ones [12,13].

### Combination of EEG and audio

This paper explores a less common multimodal approach in the MER field: combining EEG signals with audio features. While not entirely novel, this approach leverages the increasing availability of EEG datasets. Studies by Xing et al. [14] and Sawata et al. [15] have demonstrated the potential of EEG data in enhancing emotion recognition models. Thammasan et al. [16] further explored the combination of EEG and audio features in binary classification tasks for valence and arousal, concluding that audio features help stabilize the often-noisy EEG signals.

### Recent state-of-the-art

With the increasing availability of EEG data and computational resources, studies have begun to analyze EEG signals for music emotion recognition. In the past 5 years, this research has produced promising results. MER tasks typically diverge into two branches: classification and regression [17].

The classification branch is mainly focused on binary classification: determining whether the music piece is high/low arousal or high/low valence, placing the emotional impact into four quadrants. Current state-of-the-art methods exploit multiple algorithms and achieve near-perfect accuracy, as noted in Table 1. However, for the purpose of accurate detection and use of music therapy, the objectives seem too broad.

**Table 1:** Recent studies in classification of music emotion recognition.

Author	Methodology	Valence Acc.(%)	Arousal Acc.(%)
Akhand et al. (2023) [18]	Information enhancement in connectivity feature map	~91	~91
Zhang et al. (2024) [19]	Convolutional layers, improved Transformer encoder	99.23	99.17
Ahmadzadeh Nobari Azar et al. (2024) [20]	Convolutional fuzzy neural network	~98	~98

Regression gains more insight into the specific moods that the participant might feel, which makes the task more difficult. Surprisingly, few studies have done work in the regression branch, according to Table 2. More recent studies demonstrate strong linear relationships with Pearson Correlation Coefficient (PCC) (>0.75). Due to multiple input domains, PCC was excluded in this work, the R squared value was chosen instead. The square root of R squared will be compared with current state-of-the-art results for an approximate equivalence study.

**Table 2:** Recent studies in regression of music emotion recognition.

Author	Methodology	Valence PCC (%)	Arousal PCC (%)
Galvão et al. (2021) [21]	K-Nearest Neighbors (K=1, Manhattan dist.)	0.794	0.795
Javidan et al. (2021) [22]	Support Vector Regression and Multilayer Perceptron	0.67	0.67

The remainder of this paper is organized as follows: The Materials and methods section details our methodology, including the dataset used, preprocessing methods, the Machine Learning Models proposed with a description of our Convolutional Neural Network (CNN) - Multilayer Perceptron (MLP) architecture. The Results section presents and discusses the results of our models in terms of MAPE, MAE and R squared values. Finally, the Conclusion Section concludes the paper and suggests future research directions.

## Materials and methods

### Dataset

A gold standard dataset used by hundreds of studies in music emotion recognition is called the Dataset for Emotion Analysis using Physiological and Audiovisual Signals (DEAP), compiled by Queen Mary University of London, Twente University, and Geneva University [23].

It contains multimodal recordings from 32 participants, each of whom watched 40 one-minute music clips designed to evoke a range of emotional responses. After each clip, participants rated their emotional state on a 9-point scale in domain like valence and arousal.

The dataset includes both physiological signals and face video recordings, but this study mainly uses the 32 channel EEG recording as an input. It also provides the song list that the participant listens to. Though not directly provided, this study collects raw audio content and extracts features for the multimodal approach. The EEG signal was recorded at 512 Hz and downsampled to 128 Hz in the preprocessed version, which also includes artifact removal and signal normalization. This study directly accesses the preprocessed version for Python.

### Audio feature extraction

**Fig 1:**
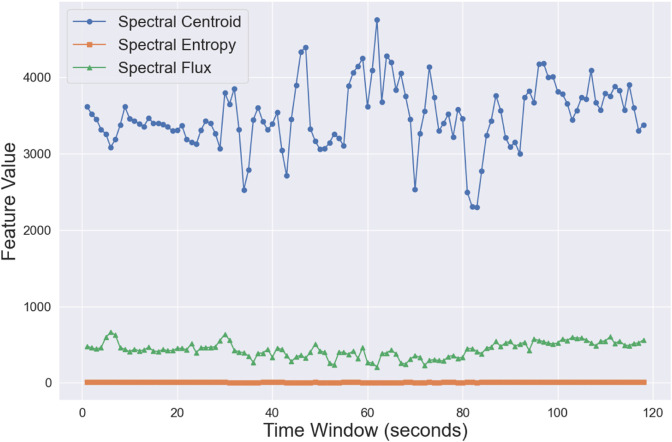
Sample feature trends for Love Story by Taylor Swift.

5 features are extracted from 1-second windows using the librosa library in Python [24]. There is 50% overlap between each window, which means a total of 118 windows for a 1-minute music piece. All features are related to timbre of audio pieces, which is shown to be more impactful towards perceived emotion [25]. They are Spectral Centroid, Centroid Spread, Centroid Skewness, Spectral Entropy, Spectral Flux. Spectral Centroid represents the center of mass of a sound spectrum; Centroid Spread measures the distribution width around the spectral centroid; Centroid Skewness shows the asymmetry of the spectral shape; Spectral Entropy measures the randomness of the spectrum. Spectral Flux captures the rate of change between successive spectra. Fig 1 is a sample demonstration on three timbre-related features in Taylor Swift’s Love Story.

### Electroencephalogram (EEG) signal preprocessing

**Fig 2:**
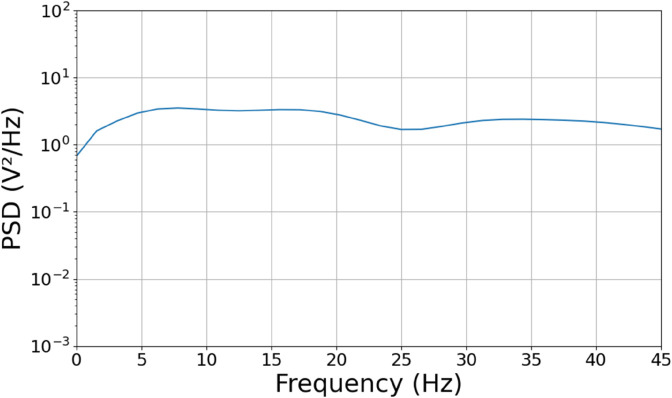
Sample preprocessed EEG recording.

The preprocessed files for Python are used in this study. However, future preprocessing is done to reduce dimensionality and remove irrelevant input for learning. The SciPy library serves this purpose [26]. The signal is filtered from 0.5Hz to 45Hz, considering conventional ranges for frequency bands in EEG studies. They include the delta band from 0.5Hz to 4Hz, the theta band from 4Hz to 8Hz, the alpha band from 8Hz to 13Hz, the beta band 13Hz to 30Hz, and the “low” gamma band from 30Hz to 45Hz. From the preprocessed signal, Power Spectral Density (PSD) is calculated using Fast Fourier Transform (FFT), Fig 2. demonstrates the processed PSD values. In this study, the frequency with the highest PSD value and the PSD value itself are considered features since they represent the dominant oscillatory activity during the recording period [27]. Considering the limitation of computational power when conducting this study and the initial commitment to provide an accessible approach to emotion prediction, no further correlations are extracted from EEG signals. This choice will sacrifice very advanced analysis on neural activities of the brain but is worthwhile for implementation and distribution in real life settings.

### Machine learning models

#### Multilayer perceptron.

A Multilayer Perceptron (MLP) Regressor is a type of artificial neural network used for regression tasks. It learns to map input features to continuous target values using multiple hidden layers of neurons with activation functions and backpropagation for training. Weights are biases that are adjusted to minimize loss functions such as Mean Absolute Error. It is one of the simplest and most common kinds of neural networks. MLP is used for learning from tabular data like timbre features that are extracted before, as it is simplistic yet powerful.

#### Convolutional Neural Network.

A Convolutional Neural Network (CNN) is another class of deep neural networks. CNNs have a distinctive architecture designed to automatically and adaptively learn spatial hierarchies of features through backpropagation. A few key components make up CNN:

Audio and EEG signals are perfect application settings for CNNs because of their ability to capture spatial and temporal features [28,29]. In audio signals, the sequential nature of sound is important, and CNNs can detect temporal patterns. Similarly, EEG signals are time series data, and CNNs can capture important temporal dynamics like oscillatory patterns. CNNs also have the capability to support multiple channels, which is needed for EEG signals.

With this hybrid structure, the model leans into the physical context and property of audio and EEG signals, truly showing how an interdisciplinary approach can help increase performance while lowering the need for computational power and data.

### Comprehensive methodology

**Fig 3:**
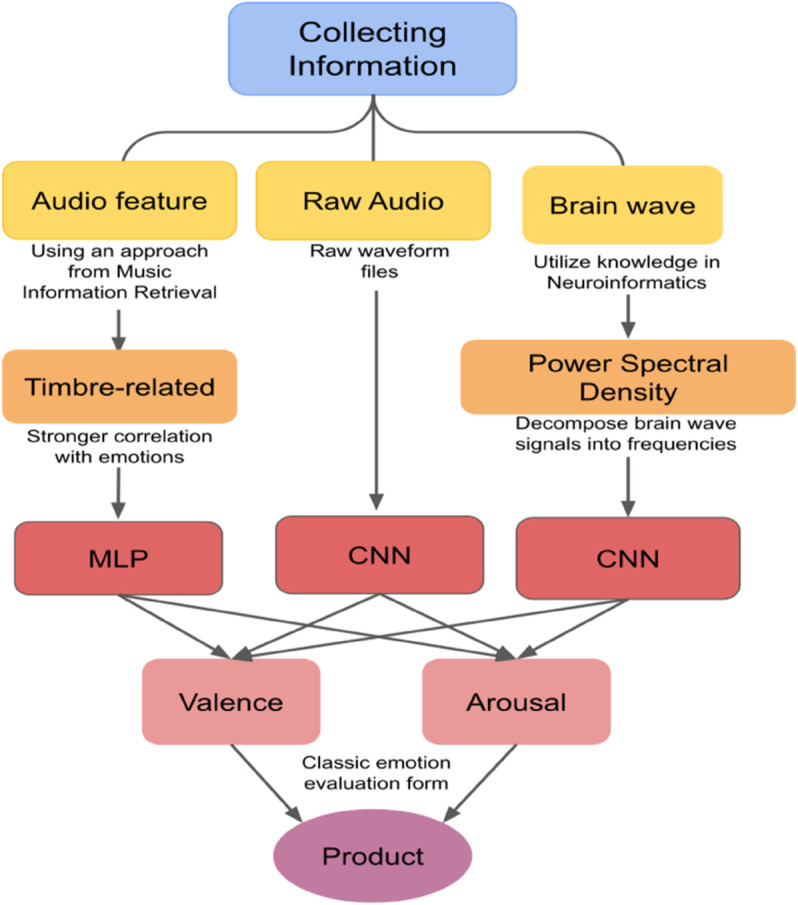
Flowchart of methodology.

There are three main input domains: extracted audio features, raw audio file and PSD values. The tabular audio features are trained with a MLP model, and sequential inputs such as raw audio file and PSD values are trained using CNN. The three models are combined to predict the final perceived emotion, evaluated by valence and arousal, a classic two-dimensional model that pictures and maps emotional states to coordinates. This method is then integrated into a publicly available product that guides users to select therapeutic music for them and improve their well-being. The workflow is shown in Fig 3.

### Training

Python 3.10 (pandas 2.2.2, pytorch 2.5.0) is used in implementing the computational result, with an NVIDIA RTX 4090 GPU as the computing device. The dataset is randomly split into training, validation and test set in a ratio of 8:1:1. The training set is used for training the model, the validation set is used for early-stopping, and the test set is used for evaluating the performance.

The architecture of the used Convolutional Neural Network (CNN) has three blocks. The first block is mainly designed for feature extraction and processing, with sequentially, a 1D Convolution layer with 8 filters and a kernel size of 9; a depth-wise 1D Convolution layer with 16 filters and a kernel size of 32; an Exponential Linear Unit (ELU) activation function to introduce non-linearity; an average Pooling layer with a factor of 4 to reduce the dimensionality; and a Dropout layer with dropout rate 0.5 to prevent overfitting. The second block, is designed for further temporal processing in the latent space. It contains a depth-wise Convolution layer with 16 filters and a kernel size of 26; a point-wise Convolution layer which helps in adjusting the number of feature maps; and an ELU activation function for non-linearity. Finally, we have the last block that is a Flattening layer to reshape the feature map of all channels for the fully connected network (FCN). After passing through the FCN that consists of two fully connected layers, one with 200 output neurons as an intermediate layer and one with 2 output neurons that maps extracted features to the final output.

The architecture of the used Multilayer Perception (MLP) is a three-layer system, with the first layer having 1278 input neurons and 1000 output neurons, the second layer having 1000 output neurons and 1000 output neurons, and the third layer having 1000 input neurons and 2 output neurons. Then, the output of the MLP and two CNNs are added together, which finally gives the predicted valence and arousal values.

## Results

We use R squared, Mean Square Error (MSE), Mean Absolute Percentage Error (MAPE) to evaluate the accuracy of the regression [30].

$$MAE=\frac{1}{n}\sum_{i=1}^{n}|y_{i}-x_{i}|$$ (1)

$$MAPE=1-\frac{100}{n}\sum_{i=1}^{n}|\frac{y_{i}-x_{i}}{y_{i}}|$$ (2)

$$R^{2}=\frac{\sum_{i=1}^{m}(x_{i}-y_{i}{)}^{2}}{\sum_{i=1}^{m}(\hat{y}-y_{i}{)}^{2}}$$ (3)

where *x*_*i*_ is defined as the *i*^*th*^ predicted value, *y*_*i*_ is defined as the *i*^*th*^ actual value and $\hat{y}$ is defined by $\hat{y}=\frac{1}{m}\sum_{i=1}^{m}y_{i}$

The multimodal approach achieves an *R*^2^ of 0.67, MAE of 1.15, MAPE of 20.15%. *R*^2^ of 0.67 suggests that the model prediction is highly correlated to the ground-truth values of valence and arousal. MAE of 1.15 is relatively low compared to the 1 to 9 scale of the valence and arousal values. MAPE is within a range that is acceptable to domain experts, suggesting that our model can reveal some patterns and correlations between the brain wave to music and the emotion. Our model performs well compared to baselines in the literature.

**Fig 4:**
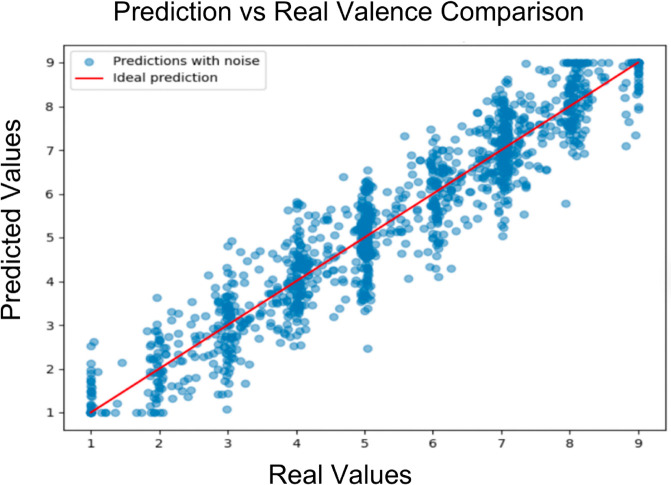
Model valence prediction visualization.

**Fig 5:**
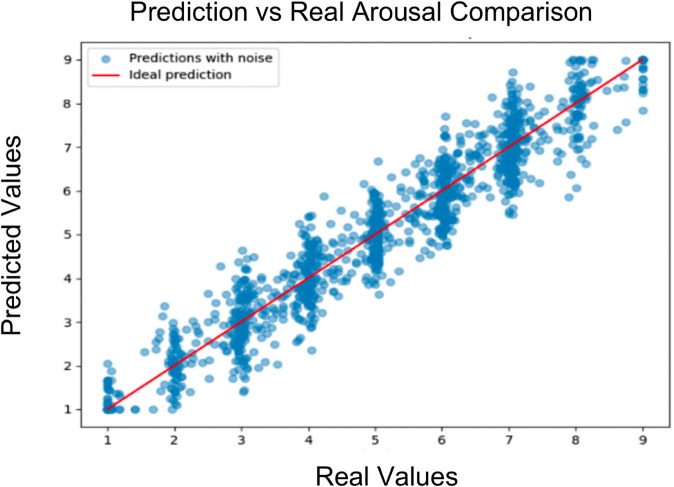
Model arousal prediction visualization.

In the following we show the predicted valence and arousal against the true values. Observations indicate that there are minimal outlier predictions and every prediction is within a reasonable bound, which shows that the model can capture the trend of whether it is high, medium, or low arousal/valence, and implies the possibility for therapeutic treatment (Figs 4 and 5).

In addition, a control ablation experiment is conducted to opt brain wave signals off as input features and combine audio features by MLP and audio waveform by CNN only. The result shows an *R*^2^ of 0.45, MAE of 1.37, MAPE of 29.97%, which is worse than adding brain wave to the modeling. This demonstrates correlations between brain wave activity and music-induced emotion.

### EEG channel contribution and correlation

**Fig 6:**
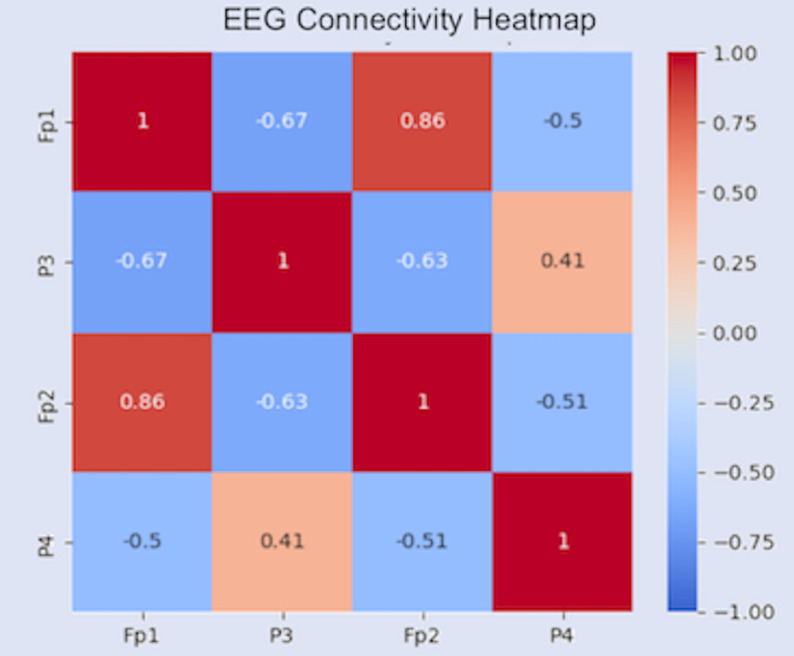
Pearson correlation between prefrontal cortex and parietal lobe.

From feature attribution in CNN, the two electrodes in the prefrontal cortex (Fp1, Fp2) and two electrodes in the parietal lobe (P3, P4) appear to be the most significant EEG channels in this prediction. This finding is also supported by neuroscience, as prefrontal cortex is the main region for cognitive control while the parietal lobe oversees sensory processing. From analyzing the Pearson correlation between the four channels in Fig 6, the two electrodes in the same region demonstrate high correlation. However, a significant negative correlation between the two regions is observed. A possible explanation for this is that regions compete for control, causing mutual inhibition in the process, but that speculation has to be verified across more subjects.

## Conclusion and future work

This study presents a novel multimodal approach to enhance music emotion recognition by integrating EEG signals with audio features, focusing particularly on predicting valence and arousal. The model achieves a MAPE value of 19.52% for valence and 22.16% for arousal, reflecting significant improvements of 10.45% and 7.81%, respectively, over predictions made using audio features alone. These results highlight the substantial contribution of EEG signals and underscore the efficacy of multimodal approaches in emotion recognition tasks, surpassing previous state-of-the-art methods. Compared to other multimodal approaches, the novelty of this study lies in utilizing EEG signals and applying it in the regression task. Previous studies mostly focus on classification of mood clusters.

Although it is hard to directly compare classification tasks and regression tasks, a regression approach such as this study can provide coordinates in the Valence-Arousal space. This captures more subtle emotions and maintains significant accuracy. A regression-based approach outperforms state-of-the-art methods. Possible explanations on why EEG signals are so effective at music emotion recognition are that EEG signals directly reflect emotional responses and are more dynamic records. Despite these advances, there are several limitations. First, the study extracts only five features from preprocessed EEG signals, suggesting that future research could benefit from incorporating a broader range of features. Additionally, the dataset used comprises 1280 data points. With the increasing availability of EEG datasets, future studies should consider using larger datasets to improve the robustness of the model. Although it is true that the DEAP dataset consists of only 32 healthy participants, this study will continue to serve its purpose even in cohorts of clinical patients. The difference in EEG signatures of emotion will be mediated if personal baselines are established by collecting the resting states of treatment subjects. In the future, work will continue with cross-dataset validation, especially data from more neurodiverse populations to enhance robustness.

In addition, the rationale behind the superior performance of MLP and CNN remains unclear and warrants further investigation. Future research should aim to address these gaps in interpretability. There are also potential applications of this model in real life. A very recent study by Hebron et al. demonstrates the potential of using auditory stimulations to modulate alpha oscillations in the brain, which is in theory the reverse engineering of our research [31]. This concept, just like ours, can be applied in clinical settings such as dementia and cognitive decline. In a more general sense, recommending appropriate music for people suffering from pain or mental breakdowns can become an alternative for traditional music therapy, making it less expensive and more accessible. This study and its results can be used to develop such a recommendation system.

The authors have already made attempts in hosting this model in cloud servers to make computation publicly accessible and easy to download and transfer for use. To further translate this work into more practical visions, this study aligns its data preprocessing to fit mainstream EEG data collection platforms such as OpenBCI, making it convenient for products from those companies to integrate into the model workflow. As the availability of EEG devices continue to increase, our model can thrive in many clinical settings. For users at home without access to EEG equipment, they can turn to our alternative model using solely audio features with MLP and audio waveform with CNN. Though not as groundbreaking as our multimodal model, this model still achieves comparable performance to the state-of-the-art and provides help in guiding users towards emotional wellness.

Also, after performing music emotion recognition tasks, EEG signals can be used and extended to other media sources, such as short videos, movies, or broadcasts and predict the emotional response.
